# Enhanced Superdense Coding over Correlated Amplitude Damping Channel

**DOI:** 10.3390/e21060598

**Published:** 2019-06-16

**Authors:** Yan-Ling Li, Dong-Mei Wei, Chuan-Jin Zu, Xing Xiao

**Affiliations:** 1 School of Information Engineering, Jiangxi University of Science and Technology, Ganzhou 341000, China; 2 College of Physics and Electronic Information, Gannan Normal University, Ganzhou 341000, China

**Keywords:** superdense coding, correlated amplitude damping channel, weak measurement, environment-assisted measurement

## Abstract

Quantum channels with correlated effects are realistic scenarios for the study of noisy quantum communication when the channels are consecutively used. In this paper, superdense coding is reexamined under a correlated amplitude damping (CAD) channel. Two techniques named as weak measurement and environment-assisted measurement are utilized to enhance the capacity of superdense coding. The results show that both of them enable us to battle against the CAD decoherence and improve the capacity with a certain probability. Remarkably, the scheme of environment-assisted measurement always outperforms the scheme of weak measurement in both improving the capacity and successful probability. These notable superiorities could be attributed to the fact that environment-assisted measurement can extract additional information from the environment and thus it performs much better.

## 1. Introduction

Superdense coding [1,2] is a simple yet surprising application of non-local properties of entangled states. The study of superdense coding is not only of great importance in quantum information theory, but also of practical significance in quantum communication. In the original proposal, the authors showed that two-bit classical information can be transmitted via a qubit and an initially shared, maximally entangled state, such as $\left(|00\rangle +|11\rangle \right)/\sqrt{2}$) [3]. This capacity is twice of the classical strategy allowed by causality. In this spirit, superdense coding has been extended experimentally to high-dimension [4] as well as multiple quantum systems [5,6,7,8,9,10], and even continuous variable systems [11]. On the other hand, the experimental realizations of superdense coding have been demonstrated in various physical platforms including linear optics [12] and nuclear magnetic resonance systems [13].

For the noise free case, it is usually believed that the maximal capacity of superdense coding is C=logd where *d* is the dimension of sender’s system [14]. In a realistic physical system, however, the achievable capacity of superdense coding remains fundamentally limited. In linear optical systems, this is confined by the fact that a complete Bell state analysis is impossible [15,16]. While in other physical systems, the unavoidable presence of noises also reduces the attainable channel capacity. In recent years, numerous attentions have been devoted to various scenarios of superdense coding under noisy channels [17,18,19,20]. Most of the earliest studies on noisy superdense coding are focused on the memoryless or uncorrelated channels. Mathematically, such an uncorrelated noise can be described by a completely positive, trace-preserving (CPTP) map ε in the quantum operation formalism [21]: an input state $\rho_{in}$ is mapped into the output state $\rho_{out}=\varepsilon \left(\rho_{in}\right)$. Consecutively using an uncorrelated channel is simply expressed as a tensor product of the CPTP map ε: $\varepsilon_{n}=\varepsilon^{⊗n}$.

Real physical systems are more or less correlated among consecutive uses [22,23]. Particularly, correlated effects become significant when the transmission rate of quantum channels is large. So, quantum memory channels have attracted growing interest in recent literatures [24,25,26,27,28,29,30]. In the correlated channels, the tensor product of ε does not valid: $\varepsilon_{n}\neq \varepsilon^{⊗n}$. References [31,32] have discussed the coding theorems of quantum capacity for long-term memory channels. Nevertheless, most of the attentions are paid to the unital memory channel, while little attentions are focused on the non-unital memory channel. The dominant noise process in many quantum information processing tasks is described by an amplitude damping channel, such as the energy relaxation. If the order of the channel’s relaxation time is comparable to or larger than that of the transmission time through the channel, then the correlated amplitude damping (CAD) noise would be remarkable. Several works have shown that the CAD noise plays a significant role in improving the entanglement [33,34] and enhancing the fidelity of teleportation [35]. The influence of CAD noise on classical capacity, quantum capacity and entanglement-assisted classical capacity is also been detailedly investigated by D’Arrigo et al. [36]. It is found that any finite amount of memory can increases the amount of reliably transmitted information with respect to the memoryless channel.

In this paper, we focus on how to improve the capacity of superdense coding under CAD channel. With the help of two recent techniques named weak measurement (WM) [37,38,39,40,41,42] and environment-assisted measurement (EAM) [43,44,45] and combining with a appropriate quantum measurement reversal (QMR), we propose two probabilistic schemes called as WM+CAD+QMR and CAD+EAM+QMR, respectively, to further enhance the capacity over the CAD channel. Interestingly, the latter scheme is comprehensively superior to the former one in the improvements of capacity as well as successful probability. Though these two schemes seem similarly, the underlying mechanisms are extremely different. Since the EAM is a post measurement, thus it extracts more information from the system and environment than the preposed WM. Our results offer an active way to improve the capacity of superdense coding under CAD channel, which is rather significant in realistic quantum communication.

The paper is arranged as follows: in Section 2, we review the key results of superdense coding in CAD channel. Then in Section 3, we propose two schemes to enhance the capacity of superdense coding in CAD channel by using WM and EAM. Moreover, in Section 4, the advantages of the CAD+EAM+QMR case are explored by comparing with the results of “WM+CAD+QMR” case. Finally, a brief summary is given in Section 5.

## 2. Superdense Coding under the CAD Channel

According to the operator-sum representation, the influence of system-environment interaction can be expressed by operators on the principal system’s Hilbert space alone. Then the evolution of the system’s state is dominated by the CPTP map $\varepsilon \left(\rho \right)=\sum_{i=0,1}E_{i}\rho E_{i}^{†}$ are Kraus operators. For the AD noise, the Kraus operators are represented as

(1) $$\begin{array}{c} E_{0}=\left(\begin{array}{cc} 1 & 0\\ 0 & \sqrt{1-\gamma } \end{array}\right),\;E_{1}=\left(\begin{array}{cc} 1 & \sqrt{\gamma }\\ 0 & 0 \end{array}\right). \end{array}$$

The parameter $\gamma \in \left[0,1\right]$ is the decoherence strength of the AD channel. For the two-qubit memoryless AD channel, identical noises act on individual qubits and the dynamical map is $\varepsilon^{⊗2}$. However, in the case of sending two qubits by successive uses of an amplitude damping channel with partial memory, the map of such a CAD channel could not be treated as a tensorial product of the individual process. In Reference [46], the authors showed that the operator-sum representation of the CAD channel is written as

(2) $$\varepsilon_{\mathrm{CAD}}\left(\rho \right)=\left(1-\mu \right)\sum_{i,j=0}^{1}E_{ij}\rho E_{ij}^{†}+\mu \sum_{k=0}^{1}A_{k}\rho A_{k}^{†},$$

here, for the two-qubit memoryless or uncorrelated AD channel, $E_{ij}=E_{i}⊗E_{j}$, while $\mu \in \left[0,1\right]$ is known as the correlation parameter of the CAD channel, μ=0 indicates that the noise is memoryless or uncorrelated, and μ=1 denotes fully correlated and the corresponding Kraus operators $A_{k}$ can be obtained by solving the correlated Lindblad equation, which gives the following formalism [47]

(3) $$\begin{array}{c} A_{0}=\left(\begin{array}{cccc} 1 & 0 & 0 & 0\\ 0 & 1 & 0 & 0\\ 0 & 0 & 1 & 0\\ 0 & 0 & 0 & \sqrt{1-\gamma } \end{array}\right),\;A_{1}=\left(\begin{array}{cccc} 0 & 0 & 0 & \sqrt{\gamma }\\ 0 & 0 & 0 & 0\\ 0 & 0 & 0 & 0\\ 0 & 0 & 0 & 0 \end{array}\right). \end{array}$$

First, we consider the behavior of superdense coding under the CAD channel. Let us suppose that the initial state is established in a maximally entangled state

(4) $$\begin{array}{c} {|\varphi \rangle }_{S}=\frac{{|00\rangle }_{S}+{|11\rangle }_{S}}{\sqrt{2}}. \end{array}$$

After it goes through the CAD channel, the pure state inevitably evolves into a mixed state which can be derived from the Equation (2)

(5) $$\begin{array}{c} \rho_{\mathrm{CAD}}=\left(\begin{array}{cccc} \rho_{11} & 0 & 0 & \rho_{14}\\ 0 & \rho_{22} & 0 & 0\\ 0 & 0 & \rho_{33} & 0\\ \rho_{41} & 0 & 0 & \rho_{44} \end{array}\right)=\left(\begin{array}{cccc} \frac{1+\bar{\mu }\gamma^{2}+\mu \gamma }{2} & 0 & 0 & \frac{\bar{\mu }\;\bar{\gamma }+\mu \sqrt{\bar{\gamma }}\;}{2}\\ 0 & \frac{\bar{\mu }\;\bar{\gamma }\gamma \;}{2} & 0 & 0\\ 0 & 0 & \frac{\bar{\mu }\;\bar{\gamma }\gamma \;}{2} & 0\\ \frac{\bar{\mu }\;\bar{\gamma }+\mu \sqrt{\bar{\gamma }}\;}{2} & 0 & 0 & \frac{\bar{\mu }\;{\bar{\gamma }}^{2}+\mu \;\bar{\gamma }\;}{2} \end{array}\right), \end{array}$$

where $\bar{\mu }=1-\mu$, $\bar{\gamma }=1-\gamma$ and $\rho_{0}={|\varphi \rangle }_{S}\langle \varphi |$.

In order to quantify the ability of superdense coding over noisy channel, the capacity $C_{CAD}^{*}$ is introduced by Hiroshima et al. [14]

(6) $$C_{CAD}^{*}=S\left({\bar{\rho_{CAD}}}^{*}\right)-S\left(\rho_{CAD}\right),$$

where ${\bar{\rho_{CAD}}}^{*}=\frac{1}{4}\sum_{i=0}^{3}\left(U_{i}⊗I\right)\rho_{CAD}\left(U_{i}^{†}⊗I\right)$ is the average density matrix of the signal ensemble $\rho_{CAD}^{*}$, in which, $\rho_{CAD}^{*}=\left(U_{i}⊗I\right)\rho_{CAD}\left(U_{i}^{†}⊗I\right)$. $S(\rho_{CAD})$ is the von Neumann entropy of $\rho_{CAD}$. $U_{i}$ are the mutually orthogonal unital transformations of dense coding for two qubits.

(7) $$U_{0}=\left(\begin{array}{cc} 1 & 0\\ 0 & 1 \end{array}\right),\;U_{1}=\left(\begin{array}{cc} 0 & 1\\ 1 & 0 \end{array}\right),\;U_{2}=\left(\begin{array}{cc} 1 & 0\\ 0 & -1 \end{array}\right),\;U_{3}=\left(\begin{array}{cc} 0 & 1\\ -1 & 0 \end{array}\right).$$

By substituting Equations (5) and (7) into (6), the capacity of superdense coding under CAD channel $C_{\mathrm{CAD}}^{*}$ can be obtained exactly by Equation (6).

(8) $$\begin{array}{c} \begin{array}{c} C_{\mathrm{CAD}}^{*}=-\left(\rho_{11}+\rho_{22}\right)\log_{2}\left(\frac{\rho_{11}+\rho_{22}}{2}\right)-\left(\rho_{33}+\rho_{44}\right)\log_{2}\left(\frac{\rho_{33}+\rho_{44}}{2}\right)+2\rho_{22}\log_{2}\left(\rho_{22}\right)\\ +\frac{\rho_{11}+\rho_{44}+\sqrt{{\rho_{11}}^{2}+{\rho_{44}}^{2}+4{\rho_{14}}^{2}-4\rho_{11}\rho_{44}}}{2\rho_{11}}\log_{2}\left(\frac{\rho_{11}+\rho_{44}\;+\sqrt{{\rho_{11}}^{2}+{\rho_{44}}^{2}+4{\rho_{14}}^{2}-4\rho_{11}\rho_{44}}}{2\rho_{11}}\right)\\ +\frac{\rho_{11}+\rho_{44}\;-\sqrt{{\rho_{11}}^{2}+{\rho_{44}}^{2}+4{\rho_{14}}^{2}-4\rho_{11}\rho_{44}}}{2\rho_{11}}\log_{2}\left(\frac{\rho_{11}\;+\rho_{44}-\sqrt{{\rho_{11}}^{2}+{\rho_{44}}^{2}+4{\rho_{14}}^{2}-4\rho_{11}\rho_{44}}}{2\rho_{11}}\right). \end{array} \end{array}$$

**Figure 1:**
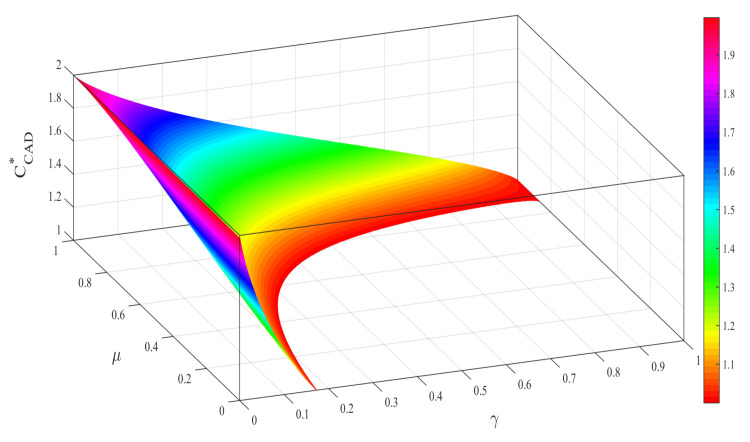
(color online) The capacity $C_{\mathrm{CAD}}^{*}$ of superdense coding as a function of the correlation parameter μ and the decoherence strength γ. The blank regions indicate the quantum advantage of superdense coding has disappeared. CAD: correlated amplitude damping.

Numerical results clearly show that the existence of correlated effect has a significant impact on the capacity $C_{\mathrm{CAD}}^{*}$. In Figure 1, the superdense coding capacity $C_{\mathrm{CAD}}^{*}$ in CAD channel is plotted as a function of correlation parameter μ and decoherence strength γ. As one might expect, $C_{\mathrm{CAD}}^{*}$ decreases with the increase of decoherence strength γ and then enters the blank region which means the quantum advantage of superdense coding disappears. While it increases with the increase of correlation parameter μ. Namely, the effects of CAD noise are twofold: The presence of AD noise indeed reduces the capacity. On the other hand, the nonlocal correlations between consecutive uses also induce the recovery of capacity. In this sense, the property of CAD channel is similar to the non-Markovian environment in open quantum systems [48].

Although the correlated effects enable us to enhance the capacity of superdense coding, the capacity is still restrained because of the destructive behavior of AD noise. Particularly, the quantum advantage of superdense coding can not be maintained in some regions. In the following, we would explore and compare two schemes for enhancing the superdense coding.

## 3. Enhanced Superdense Coding under CAD Channel

### 3.1. WM+CAD+QMR Scheme

In this section, a WM+CAD+QMR scheme is proposed to further remove the adverse effects in CAD noise. In this scheme, the initially entangled state is sequentially subjected to three steps: WM, CAD noise and QMR. The basic idea of this method stems from the fact WM is not completely destructive and can be reversed with a certain probability. Thus a preposed WM is intentionally taken to make the entangled state insensitive to the CAD noise and then a post-QMR is designed to recover the initial state. Such a procedure can be described by the following map

(9) $$\begin{array}{c} \rho_{\mathrm{WM}}=M_{\mathrm{QMR}}\left[\varepsilon_{\mathrm{CAD}}\left(M_{\mathrm{WM}}\rho_{0}M_{\mathrm{WM}}^{†}\right)\right]M_{\mathrm{QMR}}^{†}, \end{array}$$

where $M_{\mathrm{WM}}$ and $M_{\mathrm{QMR}}$ are non-unital quantum operations which are given by

(10) $$\begin{array}{c} M_{\mathrm{WM}}=\left(\begin{array}{cc} 1 & 0\\ 0 & \sqrt{1-p} \end{array}\right)⊗\left(\begin{array}{cc} 1 & 0\\ 0 & \sqrt{1-p} \end{array}\right), \end{array}$$

(11) $$\begin{array}{c} M_{\mathrm{QMR}}=\left(\begin{array}{cc} \sqrt{1-q} & 0\\ 0 & 1 \end{array}\right)⊗\left(\begin{array}{cc} \sqrt{1-q} & 0\\ 0 & 1 \end{array}\right). \end{array}$$

Note that $p,\;q\in \left[0,1\right]$ are the measurement strengths of WM and QMR, respectively. $p\in \left(0,1\right)$ indicates that the WM does not completely project the state into $|00\rangle$ or $|11\rangle$. To remove the effect of AD noise and achieve the maximal capacity, how to choose the optimal strength of the QMR is crucial. Mathematically, one can find the most optimal reversing measurement strength that gives the maximal capacity by varying *q* from 0 to 1. However, the generally analytic expression is state-dependent and not applicable in practice. In Refs. [39,40,49], according the quantum jump method [50], the authors derived a state-independent relationship of *q* which is formulated as

(12) $$q=p+\gamma \;\bar{p}.$$

in it, $\bar{p}=1-p$. Then the final state $\rho_{\mathrm{WM}}$ after the sequential WM, CAD channel and QMR is given by

(13) $$\begin{array}{c} \rho_{\mathrm{WM}}=\frac{1}{N}\left(\begin{array}{cccc} \frac{U}{2} & 0 & 0 & \frac{X}{2}\\ 0 & \frac{V}{2} & 0 & 0\\ 0 & 0 & \frac{V}{2} & 0\\ \frac{X}{2} & 0 & 0 & \frac{W}{2} \end{array}\right), \end{array}$$

where $U={\bar{p}}^{2}{\bar{\gamma }}^{2}\left(1+{\bar{p}}^{2}\bar{\mu }\;\gamma^{2}+{\bar{p}}^{2}\mu \;\gamma \right)$, $V={\bar{p}}^{3}\bar{\mu }\;\gamma \;{\bar{\gamma }}^{2}$, $W={\bar{p}}^{2}\left(\bar{\mu }\;{\bar{\gamma }}^{2}+\mu \;\bar{\gamma }\right)$, $X={\bar{p}}^{2}\bar{\gamma }\left(\bar{\mu }\;\bar{\gamma }+\mu \;\sqrt{\bar{\gamma }}\right)$. $N=\left(U+2V+W\right)/2$ is the normalization factor. Among them, $\bar{\mu }=1-\mu$, $\bar{\gamma }=1-\gamma$.

The capacity of superdense coding of the mixed state $\rho_{\mathrm{WM}}$ and the corresponding successful probability could be obtained from Equation (6). To quantify the capacity improved by the WM+CAD+QMR scheme, we introduce the capacity improvement

(14) $$C_{\mathrm{imp}}^{1}=C_{\mathrm{WM}}^{*}-C_{\mathrm{CAD}}^{*}.$$

**Figure 2:**
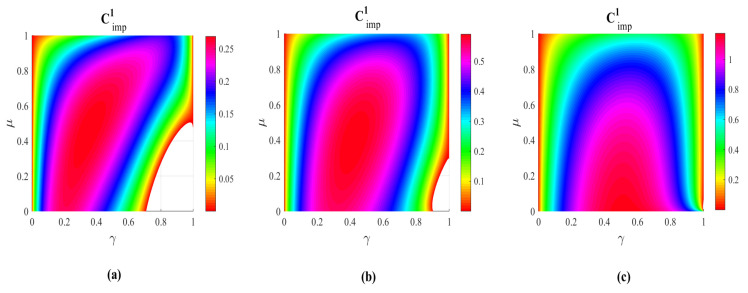
(color online) The capacity improvement $C_{\mathrm{imp}}^{1}$ as a function of the correlated parameter μ and the decoherence strength γ. From left to right, the strengths of WM p=0.1 (**a**), p=0.5 (**b**) and p=0.9 (**c**), respectively. The parameter *q* is set to $q=p+\gamma \bar{p}$.

As illustrated in Figure 2, it was found that $C_{\mathrm{imp}}^{1}$ is positive in most cases of μ and γ. The positive values of $C_{\mathrm{imp}}^{1}$ imply that the capacity of superdense coding is improved by the operations of WM and QMR. The largest improvement approaches to 1 classical bit. Even in the severely decoherence cases (i.e., γ→1), the capacity of superdense coding with the assistance of WM and QMR is always greater than that without WM and QMR as long as the WM is strong enough. Hence the quantum advantage of superdense coding is almost preserved. Since both WM and QMR operations are non-unital operations, thus the price of high capacity of superdense coding is based on the probabilistic nature of WM. The successful probability of WM+CAD+QMR scheme can be obtained as

(15) $$P_{\mathrm{WM}}^{*}=\frac{U+2V+W}{2}.$$

**Figure 3:**
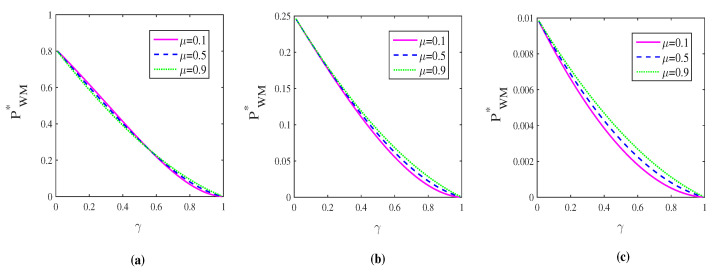
(color online) The successful probability of weak measurement (WM)+CAD+quantum measurement reversal (QMR) scheme $P_{\mathrm{WM}}^{*}$ as a function of the strength of decoherence γ for different correlated parameters μ. From left to right, the strengths of WM are p=0.1 (**a**), p=0.5 (**b**) and p=0.9 (**c**), respectively. The parameter *q* is set to $q=p+\gamma \bar{p}$.

Figure 3 shows the behavior of successful probability under WM+CAD+QMR scheme as a function of γ with different μ and *p*. Clearly, the successful probability is dependent on the correlated parameter μ, but mostly, it is determined by the measurement strength of WM. It decreases with increasing strength of WM, which means that the high capacity of superdense coding is achieved at the expense of low successful probability. The trade-off between the high capacity and low successful probability should be considered in practical situation. In Section 4, we will show that WM+CAD+QMR scheme can enhance the capacity, but the enhancement on average capacity is disappointing.

### 3.2. CAD+EAM+QMR Scheme

Though the WM+CAD+QMR scheme has been confirmed to be beneficial to the improvement of capacity, its validity is limited in the following two aspects: one is the balance between high capacity and low successful probability. The other is the effectiveness in some particular cases (i.e., the blank regions in Figure 2). It is natural to pursue a better method that can overcome these two shortages. In this section, we propose a new scheme named CAD+EAM+QMR to enhance the superdense coding in CAD noise. The distinguished difference between the WM+CAD+QMR and CAD+EAM+QMR schemes is that the WM operation is performed before CAD noise in the former, while the EAM operation is performed after the CAD noise in the later. The underlying crucial idea in the CAD+EAM+QMR scheme is to extract the information by performing a measurement on the environment followed by a QMR operation according to the environment measurement outputs. As we will show, this scheme works much better on improving the capacity and successful probability.

The CAD+EAM+QMR scheme is implemented as follows: first, we send the initial state $\rho \left(0\right)={|\varphi \rangle }_{S}\langle \varphi |$ into a CAD noisy channel. Due to the system-environment interaction, the initial state will be decohered after a period of time. An ideal detector is added to monitor the changes of environment. For the CAD noise, there are three possible outcomes (i.e., zero, one and two clicks) of the detector. We discard the result of clicks (including one or two clicks) since the reduced states of system are invertible, while the reduced state corresponding to the no click could be recovered by a QMR operation.

Now, we consider that the initial state is still given by Equation (4) and the environments are all in vacuum state. Supposing that the outcome of EAM is no click, the reduced state of the system of interest could be derived as

(16) $$\begin{array}{c} \rho^{\prime }=\left(\begin{array}{cccc} \frac{\bar{\mu }}{1+{\bar{\gamma }}^{2}}+\frac{\mu }{1+\bar{\gamma }} & 0 & 0 & \frac{\bar{\mu }\;\bar{\gamma }}{1+{\bar{\gamma }}^{2}}+\frac{\mu \sqrt{\bar{\gamma }}}{1+\bar{\gamma }}\\ 0 & 0 & 0 & 0\\ 0 & 0 & 0 & 0\\ \frac{\bar{\mu }\;\bar{\gamma }}{1+{\bar{\gamma }}^{2}}+\frac{\mu \sqrt{\bar{\gamma }}}{1+\bar{\gamma }} & 0 & 0 & \frac{\bar{\mu }\;{\bar{\gamma }}^{2}}{1+{\bar{\gamma }}^{2}}+\frac{\mu \bar{\gamma }}{1+\bar{\gamma }} \end{array}\right), \end{array}$$

To restore the initial state, a non-unital quantum operation QMR given by Equation (11) is required to perform on the qubits. Thus, the final state $\rho_{\mathrm{EAM}}$ is determined as

(17) $$\begin{array}{c} \rho_{\mathrm{EAM}}=\frac{1}{N^{\prime }}\left(\begin{array}{cccc} {\bar{q}}^{2}U^{\prime } & 0 & 0 & \bar{q}X^{\prime }\\ 0 & 0 & 0 & 0\\ 0 & 0 & 0 & 0\\ \bar{q}X^{\prime } & 0 & 0 & W^{\prime } \end{array}\right), \end{array}$$

where $U^{\prime }=\bar{\mu }/\left(1+{\bar{\gamma }}^{2}\right)+\mu /\left(1+\bar{\gamma }\right)$, $W^{\prime }=\bar{\mu }\;{\bar{\gamma }}^{2}/\left(1+{\bar{\gamma }}^{2}\right)+\mu \;\bar{\gamma }/\left(1+\bar{\gamma }\right)$, $X^{\prime }=\bar{\mu }\;\bar{\gamma }/\left(1+{\bar{\gamma }}^{2}\right)+\mu \sqrt{\bar{\gamma }}/\left(1+\bar{\gamma }\right)$. $N^{\prime }={\bar{q}}^{2}U^{\prime }+W^{\prime }$ is the normalization factor.

Based on the above results, we can calculate the capacity of superdense coding of the mixed state $\rho_{\mathrm{EAM}}$ and obtain the optimal strength of QMR numerically by varying *q* from 0 to 1. Similarly, we also define the capacity improvement between $C_{\mathrm{EAM}}^{*}$ and $C_{\mathrm{CAD}}^{*}$

(18) $$C_{\mathrm{imp}}^{2}=C_{\mathrm{EAM}}^{*}-C_{\mathrm{CAD}}^{*}.$$

**Figure 4:**
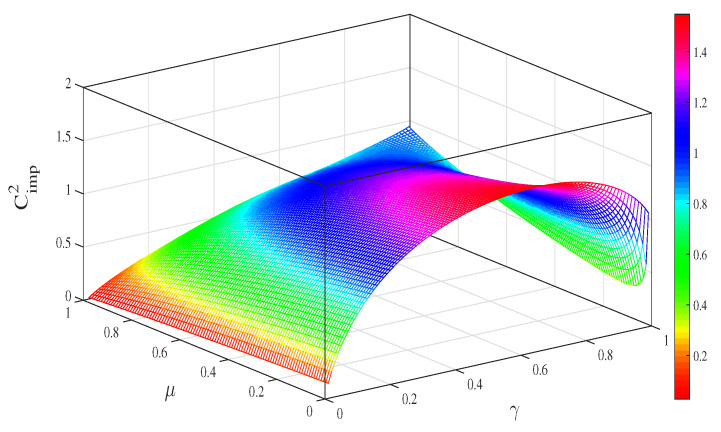
(color online) The capacity difference $C_{\mathrm{imp}}^{2}$ between $C_{\mathrm{EAM}}^{*}$ and $C_{\mathrm{CAD}}^{*}$ as a function of the memory parameter μ and the decoherence strength γ. EAM: environment-assisted measurement.

To demonstrate the power of the CAD+EAM+QMR scheme, we plot the capacity difference $C_{\mathrm{imp}}^{2}$ as a function of μ and γ in Figure 4. It is interesting to note that $C_{\mathrm{imp}}^{2}$ is always positive regardless of the values of correlated parameter μ and decoherence parameter γ. This confirms that the capacity of superdense coding in CAD noise is greatly enhanced with the assistance of EAM and QMR. Considering that the EAM and QMR are also probabilistic operations, the successful probability is given by

(19) $$P_{EAM}^{*}={\bar{q}}^{2}U^{\prime }+W^{\prime }.$$

**Figure 5:**
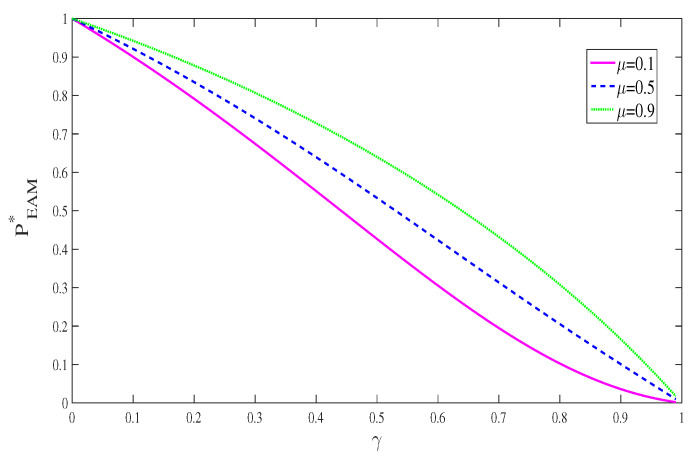
(color online) The successful probability of EAM+QMR scheme $P_{\mathrm{EAM}}^{*}$ as a function of the strength of decoherence γ for different memory parameters μ.

Figure 5 shows the behaviors of $P_{\mathrm{EAM}}^{*}$ as a function of γ with different values of μ. Though the successful probability decays with increasing decoherence parameter γ, it is improved with the increase of correlated parameter μ.

## 4. Comparison between WM+CAD+QMR Scheme and CAD+EAM+QMR Scheme

**Figure 6:**
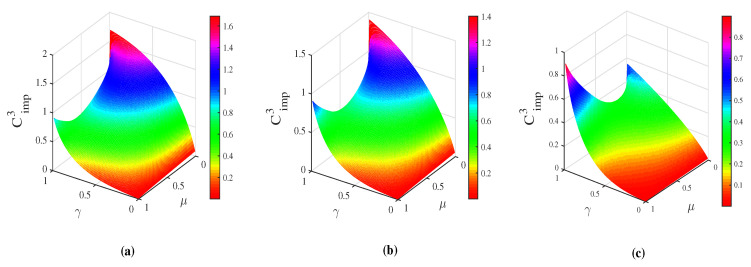
(color online) The capacity difference $C_{\mathrm{imp}}^{3}$ between $C_{\mathrm{EAM}}^{*}$ and $C_{\mathrm{WM}}^{*}$ as a function of μ and γ with p=0.1(a),0.5(b),0.9(c) in turn.

To provide a complete picture of these two schemes, we will make a detailed comparison between them from the following two aspects: capacity improvement and successful probability. Both WM+CAD+QMR and CAD+EAM+QMR schemes are devoted to improving the capacity of superdense coding in CAD noise, but the efficiency is not the same. By introducing the capacity difference $C_{\mathrm{imp}}^{3}$ which is given by

(20) $$C_{\mathrm{imp}}^{3}=C_{\mathrm{EAM}}^{*}-C_{\mathrm{WM}}^{*},$$

we can obtain an intuitive understanding of the distinction. In Figure 6, $C_{\mathrm{imp}}^{3}$ as a function of μ and γ with different strengths *p* of WM are plotted. Note that the parameter *q* has been optimized to ensure the maximal capacity. At first sight, we find that $C_{\mathrm{imp}}^{3}$ are always larger than 0 in both Figure 6a–c. This means that the CAD+EAM+QMR scheme always outperforms the WM+CAD+QMR scheme on the improving of the capacity of superdense coding in CAD decoherence.

**Figure 7:**
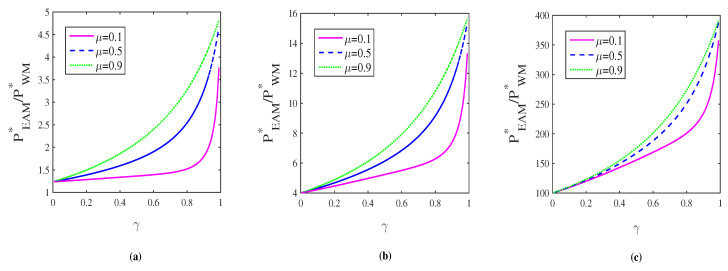
(color online) The ratio $P_{\mathrm{EAM}}^{*}/P_{\mathrm{WM}}^{*}$ as a function of γ for different μ when p=0.1(a),0.5(b),0.9(c), one after another.

Another interesting aspect worth comparing is the successful probability. In both WM+CAD+ QMR and CAD+EAM+QMR schemes, we know that a high capacity corresponds to a low successful probability. One might conjecture that the successful probability of CAD+EAM+QMR scheme is also smaller than that of WM+CAD+QMR scheme since $C_{\mathrm{EAM}}^{*}>C_{\mathrm{WM}}^{*}$. However, we argue that this intuition is not true. According to Equations (19) and (15), we define a ratio as $P_{\mathrm{EAM}}^{*}/P_{\mathrm{WM}}^{*}$ and plot the numerical results in Figure 7. Remarkably, the results show that $P_{\mathrm{EAM}}^{*}/P_{\mathrm{WM}}^{*}\geq 1$ for any given μ, γ and *p*, which means that the successful probability of CAD+EAM+QMR scheme is also larger than that of WM+CAD+QMR scheme.

**Figure 8:**
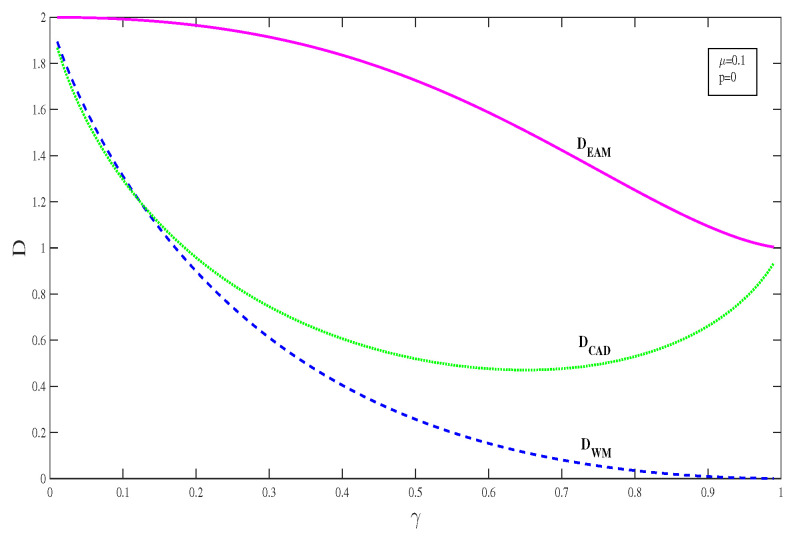
(color online) Average capacity $D=C^{*}*P^{*}$ as a function of the decoherence strength γ with μ=0.1, p=0.

In order to further verify whether the EAM and WM schemes are really effective in enhancing superdense coding under the CAD noise, we should take the combination of capacity and successful probability into consideration. Here, we introduce a quantity $D=C^{*}*P^{*}$ which quantifies the average capacity. In Figure 8, $D_{EAM}$, $D_{WM}$ and $D_{CAD}$ are plotted as a function of decoherence strength γ under CAD noise with μ=0.1, p=0. It clearly shows that $D_{WM}$ starts out a little better than $D_{CAD}$ in the weak decoherence region, but $D_{WM}$ becomes worse than $D_{CAD}$ with the increase of decoherence strength γ. However, $D_{EAM}$ is always much greater than $D_{WM}$ and $D_{CAD}$. This confirms that the EAM scheme indeed greatly improves the capacity of superdense coding in CAD noise, and significantly better than the WM scheme. We can understand this phenomenon as follows: for the WM+CAD+QMR scheme, only a part of information of the system is extracted by WM and used to correct the CAD noise. While for the CAD+EAM+QMR scheme, the post EAM not only collect the system’s information, but also gathers the additional information from the environment. Therefore, both the capacity and the successful probability can significantly surpass those obtained in WM+CAD+QMR scheme.

## 5. Discussions and Conclusions

Before conclusion, it is necessary to discuss the experimental implements of WM and EAM. References. [38,39] pointed out that WM can be achieved through a single operation, which is very important in actual experiments because of the difficulty of controlling two qubits and their coupling. In addition, the operation time is also significantly important due to the limited coherent time. As described in References. [39,40], the WM can be implemented with a Brewster angle glass plate (BAGP) for photon polarization qubit, where BAGP probabilistically refuses vertical polarization and completely transmits horizontal polarization. Those operations accurately act as the WM. It is easier to realize EAM by adding an ideal detector to monitor the environment, function as follows: the detector clicks if there is excitation (one or two) in the environment and never clicks if no excitation is detected in the environment. The no click corresponds to a success EAM operation.

Though the WM and EAM and greatly improve the capacity of superdense coding, the largest capacity is restricted to two by using entangled qubits. This limit can be surpassed by using high-dimensional entanglement, as reported in Reference [4]. The authors demonstrated a channel capacity of 2.09±0.01 with entangled qudits. However, this capacity is smaller than the limit 2.32 of owing to the fidelity of entangled qudit state and imperfect four-dimensional Bell-state measurement. It is worth to mention that our scheme could be extended to *d*-dimensional systems. For example, in Reference [41], we have shown that WM and QMR can protect qutrit-qutrit entanglement from amplitude damping decoherence. Since the capacity of superdense coding is directly dependent on the entanglement, this means that one can use WM and EAM to enhance the capacity even in the *d*-dimensional cases.

In summary, we have examined the superdense coding in the CAD noise and revealed that correlated effects are expected to increase the capacity. We also proposed two schemes named as WM+CAD+QMR and CAD+EAM+QMR to battle against CAD noise. Even though they are probabilistic strategies, the capacity is indeed drastically improved. Particularly, it is demonstrated that the CAD+EAM+QMR scheme is always better than the WM+CAD+QMR one, not only on the enhancement of capacity but also on the improvement of successful probability. The underlying factor is attributed to the difference between WM and EAM. The results obtained in this paper provide another perspective to suppress decoherence in quantum information processing.

## References

[1] Bennett C.H., Wiesne S.J. (1993). Communication via one- and two-particle operators on Einstein-Podolsky-Rosen states. Phys. Rev. Lett..

[2] Agrawal P., Pati A. (2006). Perfect teleportation and superdense coding with W states. Phys. Rev. A.

[3] Harrow A., Hayden P., Leung D. (2004). Superdense coding of quantum states. Phys. Rev. Lett..

[4] Hu X.M., Guo Y., Liu B.H., Huang Y.F., Li C.F., Guo G.C. (2018). Beating the channel capacity limit for superdense coding with entangled ququarts. Sci. Adv..

[5] Barenco A., Ekert A. (1995). Dense coding based on quantum entanglement. J. Mod. Opt..

[6] Hausladen P., Jozsa R., Schumacher B., Westmoreland M., Wootters W.K. (1996). Classical information capacity of a quantum channel. Phys. Rev. A.

[7] Bose S., Vedral V., Knight P.L. (1998). Multiparticle generalization of entanglement swapping. Phys. Rev. A.

[8] Liu X.S., Long G.L., Tong D.M., Li F. (2002). General scheme for superdense coding between multiparties. Phys. Rev. A.

[9] Grudka A., Wójcik A. (2002). Symmetric scheme for superdense coding between multiparties. Phys. Rev. A.

[10] Bruß D., D’Ariano G.M., Lewenstein M., Macchiavello C., Sen A., Sen U. (2004). Distributed quantum dense coding. Phys. Rev. Lett..

[11] Braunstein S.L., Kimble H.J. (2002). Dense coding for continuous variables. Phys. Rev. A.

[12] Barreiro J.T., Wei T.C., Kwiat P.G. (2008). Beating the channel capacity limit for linear photonic superdense coding. Nat. Phys..

[13] Wei D., Yang X., Luo J., Sun X., Zeng X., Liu M. (2004). NMR experimental implementation of three-parties quantum superdense coding. Chin. Sci. Bull..

[14] Hiroshima T. (2001). Optimal dense coding with mixed state entanglement. J. Phys. A Math. Gen..

[15] Vaidman L., Yoran N. (1999). Methods for reliable teleportation. Phys. Rev. A.

[16] Lütkenhaus N., Calsamiglia J., Suominen K.A. (1999). Bell measurements for teleportation. Phys. Rev. A.

[17] Bennett C.H., Shor P.W., Smolin J.A., Thapliyal A.V. (1999). Entanglement-assisted classical capacity of noisy quantum channels. Phys. Rev. Lett..

[18] Bennett C.H., Shor P.W., Smolin J.A., Thapliyal A.V. (2002). Entanglement-assisted capacity of a quantum channel and the reverse Shannon theorem. IEEE Trans. Inf. Theory.

[19] Shadman Z., Kampermann H., Macchiavello C., Bruß D. (2010). Optimal super dense coding over noisy quantum channels. New J. Phys..

[20] Shadman Z., Kampermann H., Bruß D., Macchiavello C. (2012). Distributed superdense coding over noisy channels. Phys. Rev. A.

[21] Nielsen M.A., Chuang I.L. (2000). Quantum Computation and Quantum Information.

[22] Holevo A.S., Giovannetti V. (2012). Quantum channels and their entropic characteristics. Rep. Prog. Phys..

[23] Caruso F., Giovannetti V., Lupo C., Mancini S. (2014). Quantum channels and memory effects. Rev. Mod. Phys..

[24] Macchiavello C., Palma G.M. (2002). Entanglement-enhanced information transmission over a quantum channel with correlated noise. Phys. Rev. A.

[25] D’Arrigo A., Benenti G., Falci G. (2007). Quantum capacity of dephasing channels with memory. New J. Phys..

[26] Plenio M.B., Virmani S. (2007). Spin chains and channels with memory. Phys. Rev. Lett..

[27] D’Arrigo A., Benenti G., Falci G., Macchiavello C. (2013). Classical and quantum capacities of a fully correlated amplitude damping channel. Phys. Rev. A.

[28] Arshed N., Toor A.H. (2006). Entanglement-assisted classical capacity of quantum channels with correlated noise. Phys. Rev. A.

[29] Benenti G., D’Arrigo A., Falci G. (2009). Enhancement of transmission rates in quantum memory channels with damping. Phys. Rev. Lett..

[30] Shadman Z., Kampermann H., Bruß D., Macchiavello C. (2011). Optimal superdense coding over memory channels. Phys. Rev. A.

[31] Bjelakovic I., Boche H., Notzel J. (2009). Entanglement transmission and generation under channel uncertainty: Universal quantum channel coding. Commun. Math. Phys..

[32] D’Arrigo A., Benenti G., Falci G. (2012). Transmission of classical and quantum information through a quantum memory channel with damping. Eur. Phys. J. D.

[33] Xiao X., Yao Y., Xie Y.M., Wang X.H., Li Y.L. (2016). Protecting entanglement from correlated amplitude damping channel using weak measurement and quantum measurement reversal. Quantum Inf. Process..

[34] Huang Z.M., Zhang C. (2017). Protecting quantum correlation from correlated amplitude damping channel. Braz. J. Phys..

[35] Li Y.L., Zu C.J., Wei D.M. (2019). Enhance quantum teleportation under correlated amplitude damping decoherence by weak measurement and quantum measurement reversal. Quantum Inf. Process..

[36] D’Arrigo A., Benenti G., Falci G., Macchiavello C. (2015). Information transmission over an amplitude damping channel with an arbitrary degree of memory. Phys. Rev. A.

[37] Korotkov A.N., Jordan A.N. (2006). Undoing a weak quantum measurement of a solid-state qubit. Phys. Rev. Lett..

[38] Katz N., Neeley M., Ansmann M., Bialczak R.C., Hofheinz M., Lucero E., O’Connell A., Wang H., Cleland A.N., Martinis J.M. (2008). Reversal of the weak measurement of a quantum state in a superconducting phase qubit. Phys. Rev. Lett..

[39] Kim Y.S., Cho Y.W., Ra Y.S., Kim Y.H. (2009). Reversing the weak quantum measurement for a photonic qubit. Opt. Express.

[40] Kim Y.S., Lee J.C., Kwon O., Kim Y.H. (2012). Protecting entanglement from decoherence using weak measurement and quantum measurement reversal. Nat. Phys..

[41] Xiao X., Li Y.L. (2013). Protecting qutrit-qutrit entanglement by weak measurement and reversal. Eur. Phys. J. D.

[42] Xiao X., Yao Y., Zhong W.J., Li Y.L., Xie Y.M. (2016). Enhancing teleportation of quantum fisher information by partial measurements. Phys. Rev. A.

[43] Zhao X., Hedemann S.R., Yu T. (2013). Restoration of a quantum state in a dephasing channel via environment-assisted error correction. Phys. Rev. A.

[44] Wang K., Zhao X., Yu T. (2014). Environment-assisted quantum state restoration via weak measurements. Phys. Rev. A.

[45] Li Y.L., Wei D.M., Zu C.J. (2019). Improving the capacity of quantum dense coding via environment-assisted measurement and quantum measurement reversal. Int. J. Theor. Phys..

[46] Yeo Y., Skeen A. (2003). Time-correlated quantum amplitude-damping channel. Phys. Rev. A.

[47] Arshed N., Toor A.H. (2013). Entanglement-assisted capacities of time-correlated amplitude-damping channel. arXiv.

[48] Breuer H.P., Petruccione F. (2002). The Theory of Open Quantum Systems.

[49] Korotkov A.N., Keane K. (2010). Decoherence suppression by quantum measurement reversal. Phys. Rev. A.

[50] Scully M.O., Zubairy M.S. (1997). Quantum Optics.

